# YTHDF2 Regulates Macrophage Polarization through NF-*κ*B and MAPK Signaling Pathway Inhibition or p53 Degradation

**DOI:** 10.1155/2022/3153362

**Published:** 2022-10-12

**Authors:** Luhui Cai, Di Li, Zhihui Feng, Xiaofei Gu, Qiong Xu, Qimeng Li

**Affiliations:** Hospital of Stomatology, Guangdong Provincial Key Laboratory of Stomatology, Guanghua School of Stomatology, Sun Yat-sen University, Guangzhou 510055, China

## Abstract

Macrophages are heterogeneous cells that can be polarized into M1 or M2 phenotype. m^6^A “reader” YTH domain family protein 2 (YTHDF2) has been the m^6^A binding protein with the highest activity, which can recognize and disturb m^6^A-containing mRNA in processing bodies to reduce mRNA stability. YTHDF2 is recently identified as an effective RNA binding protein that modulates inflammatory gene levels within inflammatory responses. However, the role of YTHDF2 in M1/M2 macrophage polarization has not been reported. We established a M1/M2 macrophage polarization model using bone-marrow-derived macrophages and found that the expression levels of YTHDF2 in M1/M2 macrophages were both elevated. YTHDF2-knockdown macrophage polarization model was then established, and through qPCR, ELISA, and FACS, we discovered that suppressing YTHDF2 encouraged M1 polarization but restrained M2 polarization. In M1 macrophages, YTHDF2 silencing had no significant effect on p53 expression; however, in YTHDF2 knockdown, M2 macrophage p53 expression was remarkably upregulated. p53 inhibitor PFT-*α* was then applied and revealed that suppressing p53 simultaneously promoted YTHDF2-silenced M1 polarization and facilitated M2 macrophage polarization. Actinomycin D assays were further utilized to examine the mRNA degradation level of different cytokines, and p53 mRNA degradation in YTHDF2-depleted M2 cells was discovered impeded. Western Blot analysis also implied that a deficit in YTHDF2 expression may activate MAPK and NF-*κ*B pathways. In this study, YTHDF2 induces M2 macrophage polarization by promoting the degradation of p53 mRNA. YTHDF2 suppresses M1 macrophage polarization by inhibiting NF-*κ*B, p38, and JNK signaling pathways, yet p53 remains unaffected in YTHDF2-silenced M1 macrophages.

## 1. Introduction

Macrophages are important innate immune cells that can trigger antimicrobial responses by identifying infection through pattern-recognition receptors [1, 2]. Their heterogeneity and malleability enable macrophages to polarize into M1 or M2 phenotypes in response to different infections [3, 4]. M1 macrophages, called the classically-activated macrophages as well, are usually induced via lipopolysaccharide (LPS) and interferon-*γ* (IFN-*γ*) and are featured by producing higher levels of surface markers CD86 and CD16/32 and excessive pro-inflammatory factors such as Interferon (IL)-1*β*, IL-6, inducible nitric oxide synthase (iNOS), and tumor necrosis factor-*α* (TNF-*α*) [5]. Upon LPS and IFN-*γ* stimulation, MAPK and NF-*κ*B pathways can be triggered to modulate the expression of many pro-inflammatory gene related to M1 polarization, which are indispensable for alleviating infections from bacteria, virus or fungus [5, 6]. M2 macrophages, also named alternatively activated macrophages, are generally featured by their upregulation of IL-10, arginase1 (Arg1), found in inflammatory zone-1 (Fizz1), transforming growth factor-*β* (TGF-*β*), dendritic cell–associated C-type lectin 1 (dectin-1), and mannose receptor (MR that encodes Mrc1, called CD206 as well) [7]. M2 cells are well known for their essential role in regulating infection, remodeling tissue, angiogenesis, and tumor progression [8–11].

N6-methyladenosine (m^6^A), methylated in adenosin's N6 position, shows the highest prevalence among internal RNA modifications in eukaryotes [8]. It has been reported and actively involved in many critical stages during the post-transcriptional course of RNA and regulates gene expression by modifying RNA processing, including localization, translation, and eventual decay [9, 10]. m^6^A deposition functions co-transcriptionally through its methyltransferases (“m^6^A writers”), comprising the catalytic subunit methyltransferase-like 3 (METTL3) and METTL14, as well as its demethylases (“erasers”) like ALKB homolog 5 (ALKBH5) and fat mass and obesity-associated protein (FTO) [11]. m^6^A modification can be “interpreted” through the binding of m^6^A “reader” proteins, like YTH-domain containing protein (YTHDC1, 2) and YTH-domain family proteins (YTHDF1-3) [12]. m^6^A is correlated with numerous biological activities, such as stem cell differentiation and pluripotency, embryogenesis, DNA damage response, and tumorigenesis [12–15]. Researches have been recently carried out to reveal the significant role of m^6^A in inflammatory responses, such as microglia inflammation, renal inflammation, and endothelial inflammation [16–18].

The m^6^A binding protein YTHDF2, belonging to the YTH domain family (YTHDF), can selectively bind m^6^A-methylated mRNA to destabilize or degrade mRNA [19]. YTHDF2 has been reported to have indispensable effect on various physiological courses, for example, neural development, cancer progression, and hematopoietic stem cell expansion [20–22]. Until recently, YTHDF2 has also been found to relate to inflammatory response progress [23]. YTHDF2 depletion within human hepatic cell carcinoma (HCC) cells or knockdown within mouse hepatocytes prompts metastasis, vascular reconstruction, and inflammation by mediating mRNA decay in cytokines that contain m^6^A [23]. Our previous study indicated that YTHDF2 regulated LPS-induced inflammatory response of macrophages through specifically mediating target mRNAs degradation [24]. As revealed by our RNA sequence results, differentially expressed genes within YTHDF2-silenced macrophages were predominantly enriched in the p53 signaling pathway (unpublished data). The transcription factor p53 is a well-known tumor suppressor and is recently reported to participate in the regulation of macrophage polarization [25–27]. In p53 deficient mice, LPS stimulation prompts pro-inflammatory cytokine production in macrophages by regulating NF-*κ*B activity [25]. Another study on p53-deficient mice revealed that in IL-4-stimulated peritoneal macrophages, M2 markers Arg1, interferon regulatory factor 4 (Irf4), Fizz1, and c-Myc were highly expressed [27]. In addition, through the mRNA analysis of p53 in the m^6^A modification database SRAMP (sequence-based RNA adenosine methylation site predictor) (http://www.cuilab.cn/sramp/), our team found that the mRNA of p53 has m^6^A modification sites (unpublished data), so it is speculated that p53 may be regulated by m^6^A and participate in the regulation of YTHDF2 on macrophage polarization. This present study focused on investigating YTHDF2 expression within M1/M2 polarization model as well as how YTHDF2 knockdown affects macrophage polarization. We further present evidence that YTHDF2 inhibited M1 polarization through the activation of MAPK and NF-*κ*B pathways while promoted M2 polarization by destabilizing *p53* mRNA.

## 2. Materials and Methods

### 2.1. Ethical Statement

This work gained approval from Ethical Review Board of Guanghua School of Stomatology of Sun Yat-sen University. Each animal experimental procedure was conducted in line with “Guide for the Care and Use of Laboratory Animals” formulated via the US National Institutes of Health. In addition, animal number adopted in this study was minimized.

### 2.2. Cell Culture and Macrophage Polarization

In this work, C57BL/6 mouse (6-8-weeks-old) were sacrificed (Animal Center of Sun Yat-sen University) and immersed them in 75% ethanol. Dissect the tibias and femurs from the body, and wash them with 90% alpha-minimal complete medium (*α*-MEM; Gibco, New York, NY, USA) including the concentration of 10% fetal bovine serum (FBS; Gibco, Carlsbad, CA, USA) with supplementation of penicillin/streptomycin. Expel the bone marrow cells with a 0.5 ml syringe containing *α*-MEM complete medium. Thereafter, cells were isolated by 24-h cultivation within *α*-MEM and 10% FBS. Besides, this work collected the suspended cells for resuspension within the *α*-MEM that contained 10% FBS and 30 ng/ml M-CSF (Sino Biological, Beijing, China). At day 6 of post-incubation under 37 °C, 95% air, and 5% CO_2_ conditions, bone-marrow-derived macrophages (BMDMs) were harvested. Logarithmic growth phase cells were utilized. After reaching 80% cell density, 0.25% trypsin/EDTA (Gibco; Thermo Fisher Scientific, Inc.) was utilized for detaching macrophages. Then, inoculation was performed (1 × 10^6^/well) into the 6-well plates. Thereafter, cells were exposed to 6-h treatment using IFN*γ* (10 ng/mL, R&D Systems, Minneapolis, MN, USA) as well as *Escherichia coli* LPS (1 *μ*g/mL, Sigma-Aldrich, St. Louis, MO, USA) or 24-h exposure to 20 ng/mL murine interleukin-4 (IL-4, Sino Biological Inc.), for the generation of M1 or M2 macrophages, separately. The untreated cells were used to be negative control.

### 2.3. YTHDF2 Small Interfering RNA (siRNA) Transfection

YTHDF2 was knocked down by siRNA transfection in macrophages. Cells (6 × 10^5^/well) were later inoculated before transfection into the 6-well plates that contained 2 ml *α*-MEM for a 24-h period. By adopting Lipofectamine ® 3000 transfection reagent (7 *μ*L, Invitrogen; Thermo Fisher Scientific, Inc.), cells reaching 70% density were exposed to transfection with 50 nM siRNA against YTHDF2 or a nontargeting siRNA control (siYTHDF2 and the negative control NC; Invitrogen, Carlsbad, CA, USA). Macrophages were subject to 24-h incubation under 5% CO_2_ and 37 °C conditions following specific protocols. The present study prepared 3 siRNA sequences for targeting mouse YTHDF2 gene.  Table 1 presents these sequences. Cells at the 70-85% transfection rate were adopted in later analysis.

### 2.4. Quantitative Real-Time PCR (qRT-PCR)

In this work, we applied TRIzol ® reagent (Invitrogen; Thermo Fisher Scientific, Inc.) in cell lysis. After extraction, Revert Aid First Strand cDNA Synthesis Kit (Takara, Tokyo, Japan) was used to synthesize cDNA from total RNA through the reverse transcription in line with specific instructions. The complementary DNA served as the template to perform PCR. Results were then detected by a LightCycler ® 480 thermal cycler. Conditions for thermal cycling included, 5-min initial denaturation under 95 °C; 10 s under 95 °C, 20 s under 65 °C, and 30 s under 72 °C for altogether 45 cycles.  Table 2 displays sequences of all primers prepared with the Primer Express Software v3.0.1 (Thermo Fisher Scientific, Inc.). *Gapdh* served as the reference for normalizing gene expression.

### 2.5. Enzyme-Linked Immunosorbent Assay (ELISA)

To analyze mouse IL-6, IL-10, TNF-*α*, and TGF-*β* contents within supernatants in line with specific protocol, the ELISA kits (R&D, Minneapolis, MN, USA) were employed. Moreover, a microplate reader was utilized for examining absorbance (OD) values at 450 nm. Sample contents were later determined based on OD values as well as standard solution concentration.

### 2.6. Flow Cytometry (FCM)

Samples were incubated with Fc receptor blocker according to the instructions for 20 minutes in dark under ambient temperature. Cells were then proceeded by antibody staining on ice for 30 min; rinse twice before resuspend them in PBS. Later, these samples were conducted FCM (Beckman, San Francisco, Calif., USA) using different antibodies to analyze samples. FITC-labeled anti-mouse CD16/32 and CD86, PE-labeled anti-mouse CD206 and F4/80 (BD Biosciences, San Jose, USA) were used to perform the analysis. At the same time, PE-labeled anti-mouse DECTIN-1 (Sino Biological Inc.) was utilized for analysis.

### 2.7. Western-Blot (WB) Assay

This work applied RIPA lysis buffer (Beyotime, Haimen, China) in combination with protease as well as phosphatase inhibitor cocktails (Cwbiotech, Beijing, China) for sample collection within a 30-min period on ice. Later, BCA protein assay (Beyotime) was conducted for measuring protein content. Subsequently, 8% SDS-PAGE was applied in separating 30 *μ*g proteins. Then, transfer on PVDF membrane was performed (Millipore, Billerica, MA, USA). After being blocked by 5% BSA for an 80-min under ambient temperature for eliminating nonspecific protein binding, the membrane was subject to overnight incubation with primary antibodies (1 : 1000) under 4 °C, including YTHDF2, p53 (Abcam, Cambridge, UK), I*κ*B*α*, p-I*κ*B*α*, IKK*α*, IKK*β*, p-IKK*α*/*β*, p38, p-p38, p65, p-p65, JNK, p-JNK, ERK, p-ERK, *β*-actin, VINCULIN, and GAPDH (Cell Signaling Technologies, Danvers, MA, USA). Membrane was rinsed TBST and further probed for a 1-h period using the 1 : 2000 diluted HRP-labeled secondary antibody (Cell Signaling Technology, Boston, MA, USA). This work employed enhanced chemiluminescence system (Millipore, Billerica, MA, USA) for protein band visualization by adopting ImageJ v1.47 software (National Institutes of Health, Bethesda, MD, USA), with GAPDH or VINCULIN being the endogenous reference.

### 2.8. RNA Stability Test and Determination of mRNA Half-Life

mRNA transcription in M1 and M2 macrophages was suppressed through exposure to 5 *μ*g/ml actinomycin D (Sigma, St. Louis, MO, USA). In addition, after being exposed to transcription suppression for 3 and 6 h, RNA samples were harvested for measuring the respective mRNA degradation. Finally, the half-life of corresponding mRNA was determined by degradation rate and level of obtained mRNA.

### 2.9. Statistical Analysis

Every assay was carried out in triplicate for 3 or more replicates. Results were shown by mean ± SD. In addition, this work utilized SPSS20.0 (SPSS Inc., Chicago, IL, USA) for One-way ANOVA and student's *t*-test in carrying out statistical analyses. Obviously, *P* < 0.05 stood for statistical significance.

## 3. Results

### 3.1. YTHDF2 Expression Increases within M1 and M2 Macrophages

To uncover m^6^A binding protein YTHDF2's role in regulating macrophage polarization, we first used bone-marrow-derived macrophages (BMDMs) to establish a M1/M2 polarization model. M0 macrophages were cells without any treatment. M0 macrophages were treated by PBS (1 *μ*g/ml) plus INF-*γ* (10 ng/ml) for 0/6/12/24 h to induce M1 macrophages and 20 ng/ml IL-4 for 0/6/12/24/48 h) to produce M2 macrophages.

**Figure 1:**
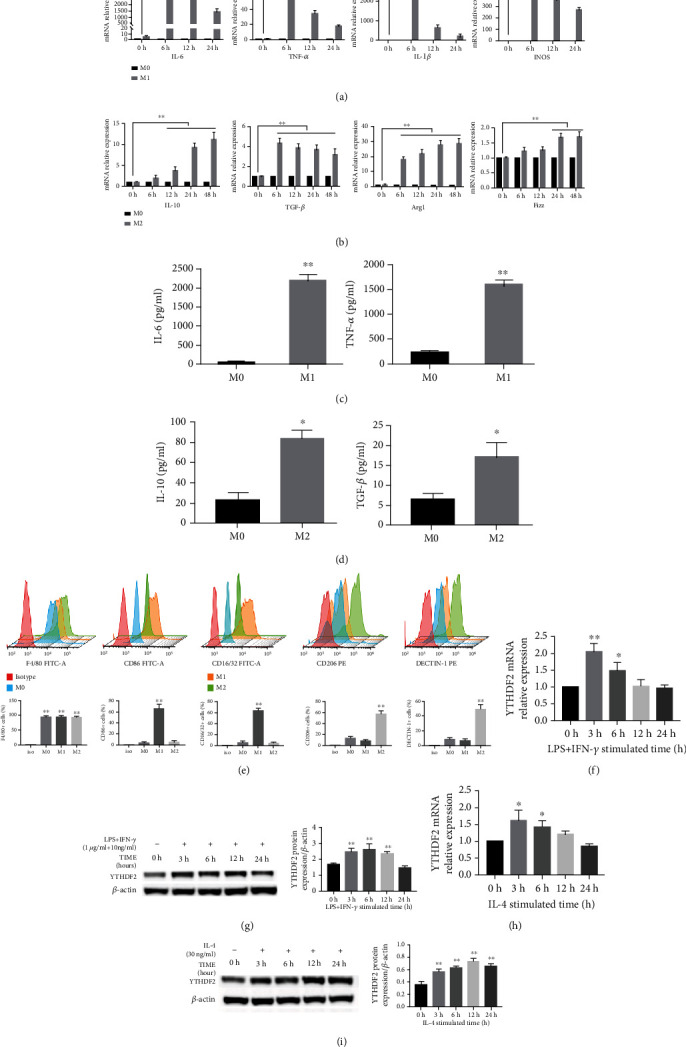
Polarization and YTHDF2 levels in M1/M2 macrophages. (a) LPS+ INF-*γ* and (b) IL-4 were utilized to treat M0 macrophages for 0, 6, 12, and 24 h, separately. *IL-1β*, *IL-6*, *IL-10*, *TNF-α*, *TGF-β*, *iNOS*, *ARG1*, and *FIZZ* levels were analyzed through qRT-PCR, with *GAPDH* being the endogenous reference. (c, d) IL-6 and TNF-*α* production in M1 cells, together with IL-10 and TGF-*β* levels within M2 cells, were detected through ELISA. (e) CD16/32, CD86, CD206, F4/80, and DECTIN-1 expression were determined through FCM in M0/M1/M2 cells. (f) *YTHDF2* mRNA level was measured through qRT-PCR following treatment of M0 cells with LPS + INF-*γ*, with *GAPDH* being the endogenous reference. (g) YTHDF2 protein expression detected through western blotting after treated M0 cells with LPS+ INF-*γ*, with *β*-actin being the endogenous reference. (h) *YTHDF2* mRNA expression quantified through qRT-PCR after treated M0 cells with IL-4, with *GAPDH* being the endogenous reference. (i) YTHDF2 protein expression measured through western blotting after treated M0 cells with IL-4, with *β*-actin being the endogenous reference. Results are demonstrated by mean ± S.E.M. (*n* = 3). ^∗^*P* < 0.05; ^∗∗^*P* < 0.01.

According to M1 marker mRNA levels displayed in Figures 1(a) and 1(b), *IL-1β*, *IL-6*, *iNOS*, and *TNF-α* expression elevated significantly after LPS/INF-*γ* stimulation at all time points, while M2 marker *ARG-1*, *TGF-β*, *IL-10*, and *FIZZ* increased with IL-4 stimulation at 12 to 48 h. The augmentation peaked at 6 h when inducing M1 polarization and at 24 h when inducing M2 polarization, which was used for further experiments. In response to 6-h LPS/INF-*γ* stimulation, IL-6 and TNF-*α* secretion significantly enhanced (Figure 1(c)), while IL-4 stimulation for 24 h prompted IL-10 and TGF-*β* secretion with significant increase (Figure 1(d)).

For further verification, this work detected cell surface markers for macrophages. After M1 stimulation, the cells presented obvious M1 phenotype with a notable growth in CD86+ cells and CD16/32+ cells, while M2 stimulation increased M2 features with an increase in DECTIN-1+ and CD206+ cells (Figure 1(e)). We further used qRT-PCR and WB assays for assessing YTHDF2 level during polarization for confirmation. The level of YTHDF2 showed a noteworthy increase after M1 (Figures 1(f) and 1(g)) and M2 stimulation (Figures 1(h) and 1(i)).

### 3.2. YTHDF2 Knockdown Promotes M1 but Inhibits M2 Polarization

**Figure 2:**
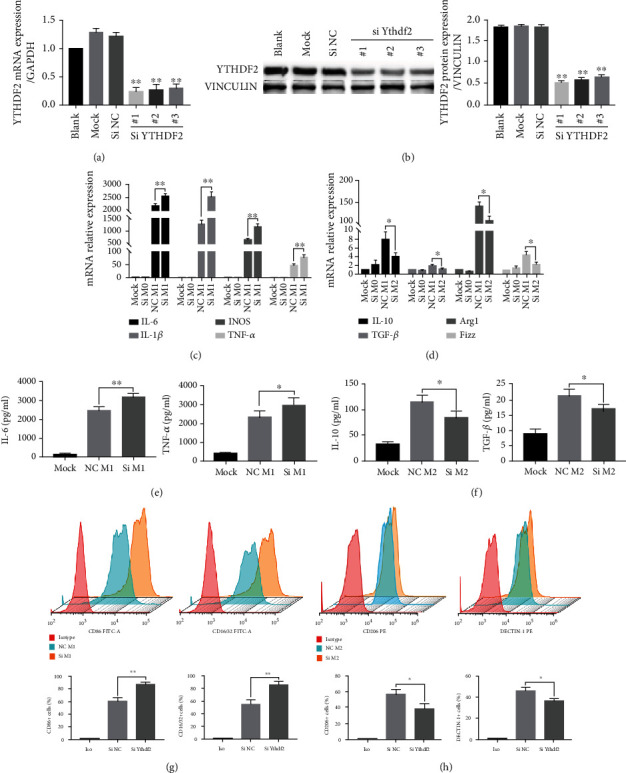
Role of YTHDF2 silencing in M1/M2 macrophage polarization. (a, b) YTHDF2 silencing efficiency in M0 macrophages was determined through qRT-PCR as well as WB. Mock: transfection reagent-transfected cells; siNC: NC-siRNA-treated cells; ^#^*n* (*n* = 1/2/3) siRNA: YTHDF2 siRNA-treated cells. (c, d) M0 macrophages were subject to transfection using YTHDF2 siRNA (Si) or NC-siRNA (NC), followed by activation using LPS+ INF-*γ* or IL-4, *IL-1β*, *IL-6*, *TNF-α*, and *iNOS* levels within M1 cells, together with *IL-10*, *TGF-β*, *ARG-1*, and *FIZZ* within M2 macrophages were determined through qRT-PCR, with *GAPDH* being the endogenous reference. (e) IL-6 and TNF-*α* production in M1 macrophages were determined by ELISA before (NC) or after YTHDF2 knockdown (Si). (f) IL-10 and TGF-*β* levels within M2 cells were determined by ELISA before (NC) or after YTHDF2 knockdown (Si). (g) CD86 and CD16/32 protein levels within M1 macrophages were detected through FACS before (NC) or after YTHDF2 knockdown (Si). (h) CD206 and DECTIN-1 levels within M2 macrophages were determined through FACS before (NC) or after YTHDF2 knockdown (Si). Data are demonstrated by mean ± S.E.M. (*n* = 3). ^∗^*P* < 0.05; ^∗∗^*P* < 0.01.

To find out YTHDF2's effect on polarization in M1/M2 macrophages, siRNAs were designed for suppressing YTHDF2 expression in M0 cells. According to Figures 2(a) and 2(b), YTHDF2 mRNA and protein expression were notably decreased after siRNA treatment. The knockdown efficiency of siYTHDF2 #1 was the highest, so we selected it to continue the following experiments.

To study the effect of YTHDF2 knockdown during polarization, YTHDF2-silienced M0 macrophages were exposed to 6-h LPS/INF-*γ* treatment to induce polarization of M1 cells or 24-h IL-4 treatment to induce polarization of M2 cells. Then, M1 and M2 markers were examined, respectively. As the result presented, *IL-1β*, *IL-6*, *iNOS*, and *TNF-α* mRNA levels elevated within siYTHDF2 M1 group (Figure 2(c)). Similarly, IL-6, TNF-*α*, CD86, and CD16/32 protein expression also increased in YTHDF2*-*knockdown macrophages (Figures 2(e) and 2(g)). Different from M1 cells, M2 markers *IL-10*, *TGF-β*, *ARG-1*, and *FIZZ* showed a significant decrease at mRNA level after IL-4 stimulation in YTHDF2-knockdown cells (Figure 2(d)). The protein levels of IL-10, TGF-*β*, CD206, and DECTIN-1 were also reduced in cells after YTHDF2 knockdown (Figures 2(f) and 2(h)). Thus, these results prompted us that YTHDF2 can inhibit M1 but encourage M2 polarization.

### 3.3. YTHDF2 Knockdown Modulates M2 Macrophage Polarization through Upregulating p53

**Figure 3:**
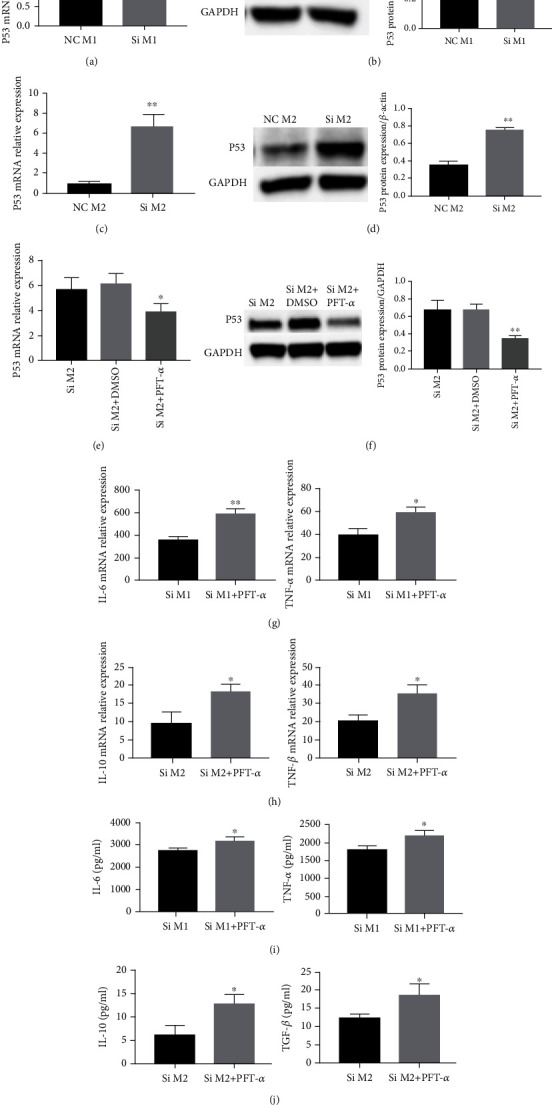
The role of p53 in YTHDF2 regulating M1/M2 macrophage polarization. (a, b) p53 mRNA and protein expression were explored through qRT-PCR as well as western blotting within M1 macrophages before and after YTHDF2 knockdown, with GAPDH being the endogenous reference. (c, d) p53 mRNA and protein expression were decided through qRT-PCR as well as western blotting within M2 macrophages before and after YTHDF2 knockdown, with GAPDH being the endogenous reference. (e, f) p53 mRNA and protein expression were detected via qRT-PCR together with WB after stimulation with PFT-*α* in YTHDF2-silenced M2 macrophages. (g, h) *IL-6*, *IL-10*, *TNF-α*, and *ARG1* mRNA levels in YTHDF2-silenced M1/M2 macrophages with or without PFT-*α* were determined through qRT-PCR, with *GAPDH* being the endogenous reference. (i, j) IL-6, IL-10, TNF-*α*, and TGF-*β* protein levels within YTHDF2-silenced M1/M2 macrophages with or without PFT-*α* were evaluated through ELISA. Results are revealed by mean ± S.E.M. (*n* = 3). ^∗^*P* < 0.05; ^∗∗^*P* < 0.01.

p53 has recently been uncovered as a regulator in macrophage polarization. To determine whether p53 has a modulating effect on macrophage polarization role in regulating M1/M2 polarization in YTHDF2-knockdown macrophages, we tested the expression of p53 in M1/M2-polarized cells, and p53 inhibitor Pifithrin-*α* (PFT-*α*) was used to inhibit p53 expression. As revealed in Figures 3(a)to 3(d), p53 mRNA and protein were increased in M2 macrophages, but remain unchanged in M1 polarization after YTHDF2 knockdown. After verifying the inhibitory effect of PFT-*α* on p53 (Figures 3(e) and 3(f)), we found that PFT-*α* promoted TNF-*α* and IL-6 mRNA levels within YTHDF2-silenced M1 macrophages, suggesting that PFT-*α* has little effect on YTHDF2-silenced M1 cells (Figure 3(g)). Meanwhile, PFT-*α* upregulated TGF-*β* and IL-10 within YTHDF2-knockdown M2 cells, indicating that PFT-*α* reversed the inhibitory effect of YTHDF2 knockdown in M2 cells (Figure 3(h)). The ELISA results were consistent with those of the qRT-PCR (Figures 3(i) and 3(j)), suggesting that the inhibition of p53 only encouraged the polarization of M2 cells after YTHDF2 knockdown. Our results suggest that YTHDF2 knockdown might impede M2 polarization by upregulating the expression of p53.

### 3.4. YTHDF2 Knockdown Increases *p53* mRNA Stability but Does Not Significantly Affect Pro-Inflammatory Cytokines mRNA Stability

**Figure 4:**
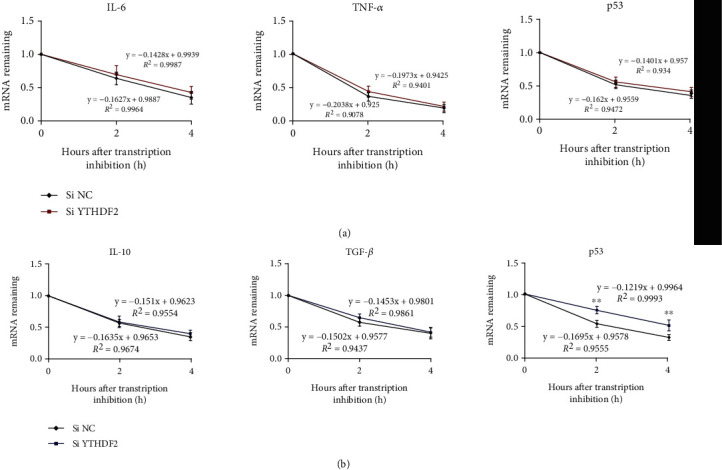
Role of YTHDF2 silencing in inflammatory cytokines and p53 mRNA stability in M1/M2 macrophages. M0 macrophages were subject to 24-h transfection using YTHDF2 siRNA (si YTHDF2) or si-NC before M1 (a) or M2 (b) stimulation. Actinomycin D (5 *μ*g/mL) was then applied for inhibiting mRNA transcription for a 0-, 2-, and 4-h period. mRNA levels of *IL-6*, *IL-10*, *TNF-α*, *TGF-β*, and *P53* were determined through qRT-PCR, with *GAPDH* being the endogenous reference. Results are indicated by mean ± S.E.M. (*n* = 3). ^∗^*P* < 0.05; ^∗∗^*P* < 0.01. The red and blue lines stand for M1 and M2-stimulated cells, respectively.

For exploring the role of YTHDF2 in regulating macrophage polarization through modulating the mRNA degradation of p53 or pro-inflammatory factors, *IL-6*, *IL-10*, *TNF-α*, *p53*, and *TGF-β* mRNA stability were measured with actinomycin D. As indicated in Figure 4, YTHDF2 knockdown promoted the stability of p53 mRNA transcript in M2 macrophages, but no significant difference was detected among the mRNA stability of the cytokines. These results indicate that YTHDF2 has no notable influence on IL-6, p53, and TNF-*α* mRNA stability of M1 cells or TGF-*β* and IL-10 of M2 macrophages; YTHDF2 silencing upregulated the expression of p53 through stabilizing its mRNA, thereby inhibiting the polarization of M2 macrophages.

### 3.5. YTHDF2 Knockdown Activates the Activity of MAPK and NF-*κ*B Pathways in M1 Macrophages

**Figure 5:**
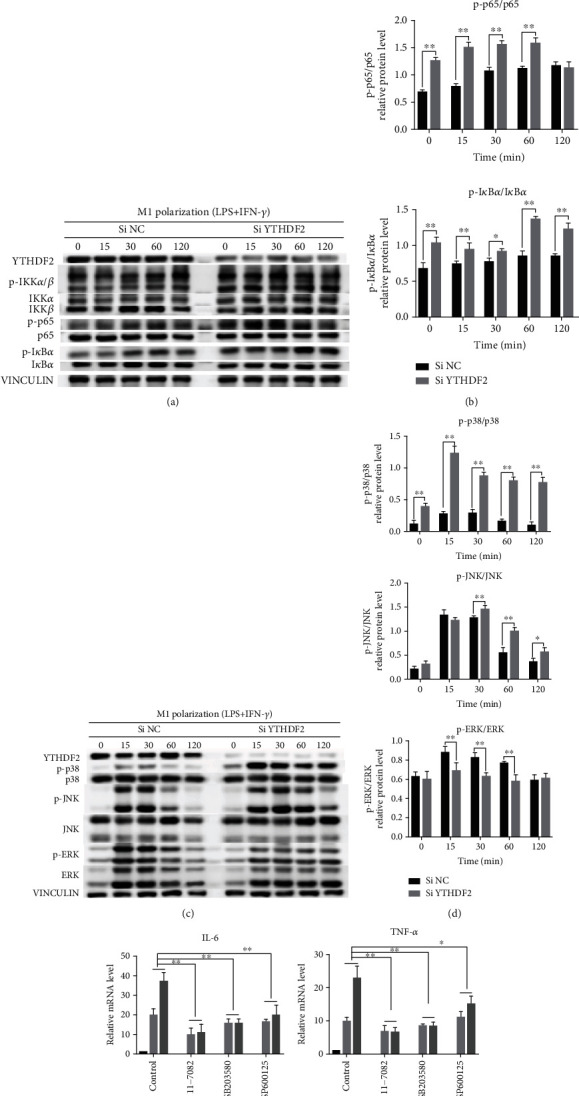
Role of YTHDF2 silencing in activation of NF-*κ*B and MAPK signalings within M1 macrophages. M0 macrophages were subject to transfection using YTHDF2 siRNAs or NC-siRNA for 24 h before 0-, 15-, 30-, 60-, and 120-min LPS+ INF-*γ* treatment. (a) p65, I*κ*B*α*, and IKK*α*/*β* phosphorylation expression within NF-*κ*B pathway were analyzed through WB, with VINCULIN being the endogenous reference. (b) Quantification of p65, I*κ*B*α*, and IKK*α*/*β* phosphorylation levels in comparison with control. (c) p38, ERK, and JNK phosphorylation levels within MAPK pathway were analyzed through western blotting. (d) Quantification of p38, JNK, and ERK phosphorylation levels in comparison with control. (e, f) M0 macrophages subject to transfection using YTHDF2 siRNA or NC-siRNA were further exposed to BAY 11-7082, SB203580, or SP600125 (inhibitors for NF-*κ*B, p38, and JNK pathways, separately) for a 2-h period, while non-treated cells served as blank control. Thereafter, LPS/IFN-*γ* was added to stimulate cells for a 6-h period. *IL-6* and *TNF-α* mRNA expression were determined through qRT-PCR, with GAPDH being an endogenous reference. Data are denoted by mean ± S.E.M. (*n* = 3). ^∗^*P* < 0.05; ^∗∗^*P* < 0.01.

MAPK and NF-*κ*B pathways have been known to be key pathways for inflammation and macrophage polarization. To discover whether YTHDF2 depletion affects these pathways within M1 macrophages, we performed WB assay for measuring p38, p65, JNK, ERK, IKK*α*/*β*, and I*κ*B*α* phosphorylation levels. As shown in our results, YTHDF2 silencing evidently induced p-p38, p-JNK, p-IKK*α*/*β*, p-p65, and p-I*κ*B*α* expression but reduced JNK phosphorylation level (Figures 5(a)-5(d)). Therefore, YTHDF2 knockdown activates NF-*κ*B pathway, as well as JNK and p38 within MAPK pathway.

For further validating how both signaling pathways affected M1 macrophage polarization in YTHDF2-silenced cells, the inhibitors NF-*κ*B (BAY 11-7082), p38 (SB203580) and JNK (SP600125) were applied sepaparately to impede the signaling. *IL-6* and *TNF-α* mRNA expression were then evaluated. According to the results, the inhibitors of NF-*κ*B, p38, and JNK reversed the increased *TNF-α* and *IL-6* mRNA levels within YTHDF2-silenced M1 cells (Figures 5(e) and 5(f)). Taken together, these results suggest that YTHDF2 suppression enhanced M1polarization by triggering the NF-*κ*B, p38, and JNK signalings.

## 4. Discussion

N6 position of adenosine (m^6^A) shows the highest prevalence among internal epigenetic modifications in mRNA [28]. Methyltransferase serves as a “writer” of m^6^A modification, demethylases work as the “eraser,” and m^6^A-selective-binding proteins act as the “reader” by selective recognizing methylated RNA to perform regulations [13, 14]. YTHDF2 is a well-known “reader” protein that targets and facilitates the degradation of m^6^A-containing RNAs [29]. Recent discoveries on YTHDF2 highlighted that YTHDF2 has a critical effect on regulating neural development, hematopoietic stem cell proliferation, cancer development, viral infection, and other physiological and pathological processes [23, 29–31].

Macrophages are heterogeneous cells endowed with great plasticity [32]. Upon exposure to different stimuli, recruited macrophages can be polarized into M1 or M2 phenotypes [3, 4, 33]. While M1 macrophages mediate innate immune responses against pathogens and activate adaptive responses through antigen processing and presentation, M2 macrophages are important in eliminating inflammation, tissue repairing, and maintaining homeostasis [34, 35]. Recently, studies in inflammatory and autoimmune diseases implicated that m^6^A modifications have regulatory roles in the activation of macrophages [36, 37]. m^6^A methyltransferase promotes M1 polarization through methylating the mRNA of *STAT1* [36]. Knockdown of demethylase FTO inhibits M1 polarization and restrains M2 activation at the same time [37]. However, the effect of m^6^A reader on macrophage activation still lingers to be elucidated.

To investigate the role of m^6^A reader YTHDF2 during macrophage polarization, BMDMs was used to establish the M1/M2 polarization system and investigated the expression of YTHDF2 after macrophage polarized. In our study, IL-4 increased the mRNA expressions of IL-10, TGF-*β*, Arg1, and Fizz in M2 cells, suggesting that M2 polarization model was established successfully. However, the changing expression levels of each inflammatory factor were not consistent at different time points. Secreted cytokines can bind to different receptors to induce activation of an intracellular cascade of signal transduction, which leads to various cellular responses. However, not all cells within an organism are identical. They differ in the amount of proteins involved in signal transduction. These differences shape cellular communication and responses to intracellular signaling. So, there might be a negative regulation responsible for the expression level of TGF-*β* to begin to reduce after a sharp increase [38]. After confirming the activation of M1/M2 phenotypes, YTHDF2 mRNA and protein expression were determined. As presented in our result, YTHDF2 expression increased significantly within M1 and M2 polarized cells, indicating that YTHDF2 might be involved in regulating macrophage polarization.

YTHDF2 can selectively recognize m^6^A to regulate mRNA degradation [15, 19]. Studies have shown that YTHDF2 enhances the capacity of self-renewal of the leukemia stem cells and neural stem/progenitor cells by suppressing the stability of multiple mRNAs critical for cell expansion [39]. YTHDF2 depletion in zebrafish embryos slows down the decline of maternal mRNAs that been m^6^A-modified and impedes the cell cycle, thereby restraining the growth development during vertebrate embryogenesis [29]. In the study of infectious diseases, YTHDF2 upregulation promotes HIV-1 and HBV levels as well as viral replication ability [40, 41]. Most recently, a study by our team found that YTHDF2 negatively regulates the mRNA expression levels of MAP2K4 and MAP4K4 via destabilizing their mRNA transcripts, which inhibits the inflammatory response in LPS-stimulated inflammatory reactions [24]. For exploring YTHDF2's effect on macrophage polarization, YTHDF2 expression was silenced in BMDMs, and M1 and M2 markers levels were examined. IL-1*β*, IL-6, iNOS, TNF-*α*, CD86, as well as CD16/32 levels were upregulated within YTHDF2-silenced M1 cells. Meanwhile, in YTHDF2-silenced M2 cells, the secretion of M2 markers IL-10, TNF-*α*, ARG-1, FIZZ, CD206, and DECTIN-1 experienced a significant reduction. Therefore, these findings demonstrated that the expression of YTHDF2 is increased in both M1/M2 cells and that YTHDF2 might have different roles during the orientation of macrophages, with YTHDF2 inhibiting M1 but promoting the M2 phenotype.

Our preliminary RNA sequence results found that genes differentially expressed in YTHDF2-silenced macrophages were mainly enriched in the p53 signaling pathway. The gene p53 is the most common tumor suppressor gene in human cancer [42, 43]. It functions as a crucial regulatory node through monitoring the expression of genes associated with metabolism, cell cycle arrest, and apoptosis [44–46]. Studies in cancer have found that inflammation is a vital aspect when it comes to determining its predisposition [47, 48] and p53 has recently been discovered working as a regulator in various inflammatory diseases [25, 26, 27, 49]. As a guardian of homeostasis, p53 plays a protective role by inhibiting the local inflammation of rheumatoid arthritis patients and collagen-induced osteoarthritis in mice [49]. p53 also controls immunity by directly impeding the activation of p65 promoter in the NF-*κ*B signaling and negatively regulating its transcriptional expression of its downstream genes IL-6, Cox-2, and Nos2 [25]. Recent studies found that p53 can regulate macrophage polarization [26, 27, 50–52]. Macrophages lacking p53 promoted the responses to LPS stimulation, producing more pro-inflammatory M1 marker genes, like IL-6, TNF-*α*, and MIP-2 [26]. When marrow-derived macrophages activated towards to M2 phonotype, cells display endogenous p53 activity and the p53 activation can in turn inhibit expression of M2 genes [27]. The p53 activator Nutlin-3a in bone marrow-derived macrophages can reduce the expression of M2 subtype [51]. To investigate whether p53 involved in the activation of YTHDF2-deficent macrophages, we examined the expression of p53 in macrophages after YTHDF2 knockdown. As shown in the result, YTHDF2 silencing did not significantly affect p53 level within M1 cells; however, p53 increased within YTHDF2-silenced M2 cells. To further determine the role of p53, the p53 inhibitor Pifithrin-*α* (PFT-*α*) was used to suppress the p53 expression in YTHDF2-knockdown cells. According to the above findings, PFT-*α* pretreatment further increased TNF-*α* and IL-6 levels within YTHDF2-knockdown M1 cells, but the reduction of TGF-*β* and IL-10 within YTHDF2-knockdown M2 subtype was reversed. Collectively, YTHDF2 promoted M2 polarization by regulating p53, but did not repress the M1 polarization directly through p53.

Accumulating evidences suggest that m^6^A may regulate the stability of the RNA through the effects of YTHDF2 [29]. The C-terminal YTH domain (YTHDF2-C) of YTHDF2 can selectively bind m^6^A, whereas P/Q/N enriched N-terminal region promote target mRNA migration into cytoplasmic foci (P bodies) while recruiting RNA import of CCR4-NOT deadenylase complex [53]. To investigate the role of YTHDF2 in destabilizing the gene transcripts of related cytokines in macrophage polarization, we measured the stability of *IL-6*, *IL-10*, *TNF-α*, *p53*, and *TGF-β* mRNAs in YTHDF2-depletion cells. According to our results, YTHDF2 silencing promoted p53 mRNA stability in M2 macrophages but had little effect on p53 stability in M1 macrophages. We further analyzed and predicted p53 mRNA on the m^6^A database SRAMP and found that p53 mRNA may have 11 m^6^A modification sites, while among other polarization-related factors, only IL-6 and ARG1 have a few m^6^A modification sites, which contains 3 and 1, respectively. This might explain why loss of YTHDF2 exhibited no detectable effect on the mRNA stability of TNF-*α* and IL-6 in M1 cells or TGF-*β* and IL-10 in M2 cells. Therefore, we concluded that YTHDF2 may encourage M2 polarization by promoting the degradation of p53 mRNA, but inhibit M1 polarization leaving the mRNA stability of inflammatory factors unaffected.

MAPK and NF-*κ*B pathways have been identified as key pathways for inflammation and M1 macrophage polarization [54–56]. Recent studies discovered that YTHDF2 upregulation can inhibit ERK and MEK activation within liver cancer cells [23]. For verifying whether YTHDF2 regulates M1 polarization by deactivating MAPK and NF-*κ*B pathways, we detected some critical molecules related to the above pathways for their phosphorylation levels after silencing YTHDF2. In our results, IKK*α*/*β*, p65, I*κ*B*α*, p38, and JNK phosphorylation levels significantly elevated after YTHDF2 silencing, but ERK phosphorylation level was suppressed. Studies have found that ERK1/2 mainly function during cell growth, proliferation, and differentiation, while p38 and JNK play essential roles in inflammation, cytokine production, and apoptosis. Besides, the activation of ERK can be suppressed by JNK and p38 kinase. These might explain the inactivation of ERK in YTHDF2-knockdown M1 macrophages. Furthermore, we inhibited the above pathways to validate their effects on regulating M1 markers' expression. Our results stated that inhibitors of NF-*κ*B, p38, and JNK pathways downregulated IL-6 and TNF-*α* levels in YTHDF2-depleted M1 cells, confirming that YTHDF2 impeded macrophage M1 polarization by inhibiting NF-*κ*B, p38, and JNK signaling pathways.

**Figure 6:**
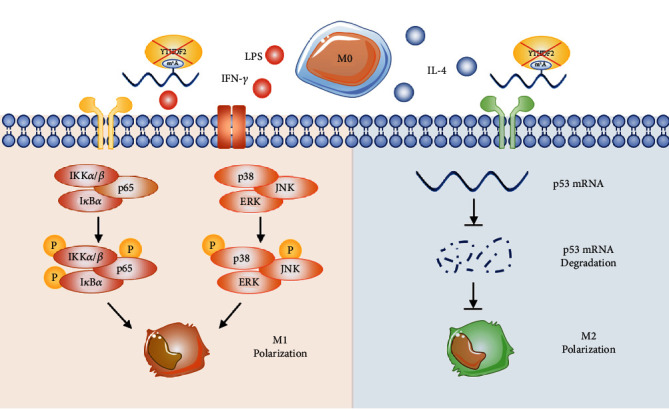
Role of m6A “reader” YTHDF2 in polarization of M1/M2 macrophages. YTHDF2 suppression promotes polarization of M1 cells via MAPK and NF-*κ*B pathway activation. Moreover, depleting YTHDF2 stabilizes p53 mRNA and upregulates its expression, thereby inhibiting the polarization of M2 macrophages.

To sum up, the m^6^A reader YTHDF2 increased its expression within M1 and M2 polarized cells. YTHDF2 silencing encouraged M1 but diminished M2 macrophage polarization. Mechanistically, silencing YTHDF2 promoted the stability of *p53* mRNA and its expression level, thereby impeding M2 macrophage polarization; YTHDF2 depletion facilitated M1 polarization by triggering both MAPK and NF-*κ*B pathways (Figure 6). In this study, YTHDF2 was identified with its regulatory role during M1/M2 polarization. Our present research on m^6^A reader YTHDF2 may offer an alternative approach for the understanding of macrophage plasticity and probably a newfound target for treating inflammatory diseases.

## Figures and Tables

**Table 1:** siYTHDF2 sequences for transcription (5′-3′).

siRNA	Sequences (5′-3′)
#1 siRNA	CCAUGAUUGAUGGACAGUCAGCUUU
#2 siRNA	CCCAGUGGGAUUGACUUCUCAGCAU
#3 siRNA	GGGUGGAUGGUAAUGGAGUAGGACA

**Table 2:** Primers used for the analysis of mRNA levels by qRT-PCR.

Gene	Forward primer (5′-3′)	Reverse primer (5′-3′)
*YTHDF2*	ATAGGAAAAGCCAATGGAGGG	CCAAAAGGTCAAGGAAACAAAG
*IL-6*	CTGCAAGAGACTTCCATCCAG	AGTGGTATAGACAGGTCTGTTGG
*IL-10*	AGCCTTATCGGAAATGATCCAGT	GGCCTTGTAGACACCTTGGT
*IL-1β*	CTTTGAAGTTGACGGACCCC	GCTTCTCCACAGCCACAATG
*Arg-1*	GCTGGGAAGGAAGAAAAAGGC	TGCCGTGTTCACAGTACTCT
*TNF-α*	CCACCACGCTCTTCTGTCTA	GGTCTGGGCCATAGAACTGA
*TGF-β*	TACATGCTCTAACTGAAGGGGA	TTGGATTTCTTCGCAAATGGTTC
*Fizz*	CAGAAGGCACAGCAGTCTTG	GGGTATTAGCTCCTGTCCCC
*p53*	GGCGTAAACGCTTCGAGATG	AAGGCTTGGAAGGCTCTAGG
*iNOS*	CGGGTTGAAGTGGTATGCAC	CACAGCCACATTGATCTCCG
*GAPDH*	GCAAAGTGGAGATTGTTGCC	TGGAAGATGGTGATGGGCTT

## Data Availability

All data utilized in the present work can be obtained from corresponding author upon reasonable request.
